# PFC/M1 activation and excitability: a longitudinal cohort study on fatigue symptoms in healthcare workers post-COVID-19

**DOI:** 10.1186/s12967-024-05319-z

**Published:** 2024-08-05

**Authors:** Tao Han, Chunqiu Dai, Ying Liang, Xiaodong Lin, Ming Gao, Xinyu Liu, Xiangbo Wu, Yuheng Lu, Xiao Xi, Fei Tian, Chenguang Zhao, Xiaolong Sun, Hua Yuan

**Affiliations:** 1 Department of Rehabilitation Medicine, Xijing Hospital, Air Force Medical University (Fourth Military Medical University), Xi’an, 710032 PR China; 2 Department of Health Statistics, Air Force Medical University (Fourth Military Medical University), Xi’an, 710032 PR China; 3 Lintong Rehabilitation and Convalescent Centre, Xi’an, 710600 PR China

**Keywords:** COVID-19, Long COVID, Fatigue, Healthcare Workers, Functional near-infrared spectroscopy, Transcranial magnetic stimulation

## Abstract

**Background:**

Fatigue is one of the most common neurological symptoms reported post coronavirus disease 2019 (COVID-19) infection. In order to establish effective early intervention strategies, more emphasis should be placed on the correlation between fatigue and cortical neurophysiological changes, especially in healthcare workers, who are at a heightened risk of COVID-19 infection.

**Methods:**

A prospective cohort study was conducted involving 29 COVID-19 medical workers and 24 healthy controls. The assessment included fatigue, sleep and health quality, psychological status, and physical capacity. Functional near-infrared spectroscopy (fNIRS) was employed to detect activation of brain regions. Bilateral primary motor cortex (M1) excitabilities were measured using single- and paired-pulse transcranial magnetic stimulation. Outcomes were assessed at 1, 3, and 6 months into the disease course.

**Results:**

At 1-month post-COVID-19 infection, 37.9% of patients experienced severe fatigue symptoms, dropping to 10.3% at 3 months. Interestingly, the remarkable decreased activation/excitability of bilateral prefrontal lobe (PFC) and M1 were closely linked to fatigue symptoms after COVID-19. Notably, greater increase in M1 region excitability correlated with more significant fatigue improvement. Re-infected patients exhibited lower levels of brain activation and excitability compared to single-infection patients.

**Conclusions:**

Both single infection and reinfection of COVID-19 lead to decreased activation and excitability of the PFC and M1. The degree of excitability improvement in the M1 region correlates with a greater recovery in fatigue. Based on these findings, targeted interventions to enhance and regulate the excitability of M1 may represent a novel strategy for COVID-19 early rehabilitation. Trial registration: The Ethics Review Committee of Xijing Hospital, No. KY20232051-F-1; www.chictr.org.cn, ChiCTR2300068444.

**Supplementary Information:**

The online version contains supplementary material available at 10.1186/s12967-024-05319-z.

## Background

The outbreak of coronavirus disease 2019 (COVID-19) has resulted in the loss of over 6 million lives and infected more than 600 million people [1], posing significant global health challenges. Despite the WHO no longer categorizing COVID-19 as a public health emergency of international concern, it continues to attract the attention of scientists. Recently, the JN.1 variant has been growing rapidly in several countries, causing ongoing surveillance by the WHO. Healthcare workers, due to the nature of their work, continue to face a heightened risk of COVID-19 infection. A survey of 84,000 medical workers revealed that 54.4% contracted the virus at medical posts [2]. Fatigue has emerged as a prevalent symptom after COVID-19 [3], affecting 42% of frontline healthcare workers for varying durations, from one week to over three months [4]. As the COVID-19 pandemic endures, medical workers confront ongoing challenges, including a high infection rate, the prospect of repeated infections, and threats to medical security. Beyond fatigue, healthcare workers grapple with psychological challenges. A meta-analysis on mental health revealed that 29% of Chinese medical staff experienced depression, 27% faced anxiety, and 40% encountered sleep problems during the COVID-19 pandemic [5]. Notably, higher depression scores can exacerbate fatigue symptoms and increase the susceptibility to COVID-19 [6, 7]. Working in a state of fatigue and emotional distress significantly jeopardizes medical safety. Unfortunately, the precise mechanism underlying “Long COVID” remains unclear, hindering the development of effective intervention to quickly relieve COVID-related fatigue.

Some researchers propose that post-COVID-19 fatigue symptoms may be linked to virus-induced neuroinflammation and dysfunction in neurotransmitter synthesis [8]. Understanding brain functional changes and their correlation with fatigue symptoms may guide early rehabilitation efforts. Functional near-infrared spectroscopy (fNIRS) can detect neurovascular changes in the cerebral cortex, reflecting brain activation levels. Paired-pulse and single-pulse transcranial magnetic stimulation (ppTMS and spTMS) enable exploration of excitatory changes in the primary motor cortex (M1) region and the corticospinal tract. By these technologies, Ortelli P et al. [9] found that fatigue symptoms in mild COVID-19 patients were associated with decreased excitability in the M1 region of the dominant hemisphere (DH). However, it is known that both hemispheric M1 are mutually inhibiting each other based on the interhemispheric inhibition model theory [10]. In addition, the prefrontal cortex (PFC) regulates higher emotions and can also influence fatigue symptoms. It remains unclear whether excitability changes in bilateral M1 regions and other brain areas affect post-COVID-19 fatigue symptoms.

This study aims to observe the severity and duration of fatigue in healthcare workers after COVID-19, comparing the effects of SARS-CoV-2 infection on the PFC, premotor cortex (PMC), M1, and primary somatosensory area (S1) between healthy controls and COVID-19 healthcare workers. Importantly, we sought to provide ideas for improving fatigue symptoms through targeted treatment for healthcare workers at high SARS-CoV-2 exposure risk.

## Methods

### Study design

**Fig. 1:**
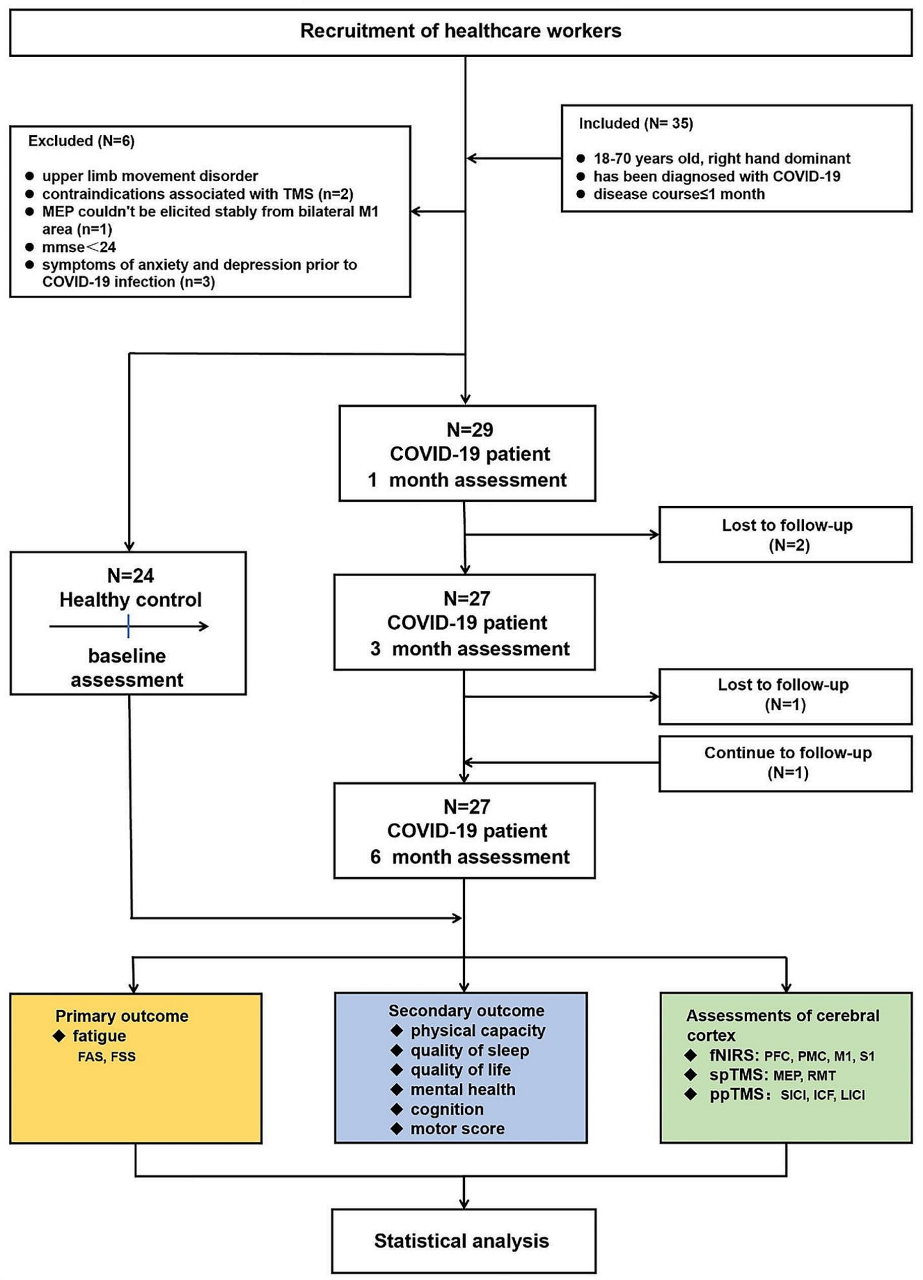
Flow chart

This prospective cohort study exclusively enrolled healthcare workers, comprising both COVID-19 patients and age/sex-matched healthy controls. Evaluation encompassed fatigue, sleep and health quality, psychological status, and cognition through questionnaires. Physical capacity was assessed using the six-minute walk test (6MWT). fNIRS detected activations of PFC, PMC, M1, and S1. spTMS measured changes in resting motor threshold (RMT) and motor evoked potential (MEP). ppTMS assessed short-interval intracortical inhibition (SICI), intracortical facilitation (ICF), and long-interval intracortical inhibition (LICI) in bilateral M1 regions. COVID-19 patients were evaluated repeatedly at 1 month (1 m), 3 months (3 m), and 6 months (6 m) post-infection. The healthy control group was measured once. The experimental workflow is depicted in Fig. 1. To ensure the accuracy and reliability, data for all participants were collected at the same time each morning. Clinical assessments and neurophysiological measurements were independently performed by dedicated and experienced technicians blinded to each other. For the assessments of all participants at different time points, we used fixed equipment to ensure the consistency of data collection before and after.

This study was granted by the Ethics Review Committee of Xijing Hospital (No. KY20232051-F-1), and it was registered in the Chinese Clinical Trial Registry (ChiCTR2300068444). Our study was conducted based on the Declaration of Helsinki. Each participant provided written informed consent.

### Sample size calculation

The sample size calculation was conducted via PASS 11.0 software based on the results of the Fatigue Assessment Scale (FAS) in our pre-experiment, which showed that about 18% of 11 healthy controls had symptoms of fatigue (FAS>21, P1 = 0.18), and about 60% of 10 COVID-19 patients had symptoms of fatigue (FAS>21, P2 = 0.6). Other parameters: α = 0.05 (two tails), power (1–β) = 90%. Therefore, a minimum sample size of 24 COVID-19 patients and 24 healthy controls were obtained. Considering the dropout rate in the cohort study, the number of patients was increased by 10%, and a minimum total of 51 participants was needed.

### Participants

#### Recruitment

In February 2023, twenty-nine medical workers diagnosed with SARS-CoV-2 infection and twenty-four SARS-CoV-2 uninfected age/sex-matched medical workers were enlisted for participation in this cohort study. The collection of questionnaires and neurophysiologic data took place at the Department of Rehabilitation Medicine in Xijing Hospital.

#### Inclusion and exclusion criteria

Inclusion criteria were: (a) age of 18–70 years old, right hand dominant; (b) healthcare workers diagnosed with mild COVID-19 with disease course less than 1 m. COVID-19 diagnosis and clinical classification were referred to the *Diagnosis and Treatment Protocol for Novel Coronavirus Pneumonia* (Trial Version 10) [11]. Exclusion criteria were: (a) upper limb fracture, arteriovenous fistula, or other diseases leading to upper limb movement disorder; (b) contraindications associated with TMS identified using the Transcranial Magnetic Stimulation Adult Safety Screen questionnaire [12]; (c) MEP couldn’t be elicited stably from bilateral M1 area; (d) cognitive impairment (Mini-Mental State Examination (MMSE) score<24); (e) symptoms of anxiety and depression prior to COVID-19 infection.

Inclusion criteria for healthy controls stipulated the absence of evidence of SARS-CoV-2 infection, confirmed by a negative result on the SARS-CoV-2 nucleic acid test. The remaining criteria for healthy controls were identical for the COVID-19 patient group.

## Outcomes measures

### Primary outcomes

#### FAS and fatigue severity scale (FSS)

Fatigue levels were evaluated using the FAS and the FSS. A higher FAS score reflects increased fatigue, with a total score exceeding 21 indicating the presence of fatigue [13]. On the other hand, the elevated FSS scores suggest more significant fatigue, and a mean score of ≥ 5 is indicative of clinically significant fatigue [14].

### Secondary outcomes

#### 6MWT

For the assessment of physical capacity, the 6MWT was employed. This test was conducted in a 30-meter outdoor hallway, instructing participants to walk as briskly as possible.

#### Pittsburgh Sleep Quality Index (PSQI)

PSQI was used to evaluate sleep quality, with higher scores indicating poorer sleep quality. A total score of ≥ 8 suggests poor sleep quality.

#### European quality of life five-dimension five-level scale (EQ-5D-5L)

Health-related quality of life was measured using the EQ-5D-5L. The EQ-5D-VAS score and EQ-5D index, obtained from the questionnaire, reflect the health status of the population.

#### Hamilton Anxiety Scale (HAMA) and Hamilton Depression Scale (HAMD)

The severity of anxiety symptoms was assessed using the HAMA, where a total score ≥ 7 indicates possible anxiety. The HAMD, a widely used tool in clinical depression evaluation, was employed to assess depression. A total score > 7 indicates potential depression.

#### MMSE

The MMSE comprises five cognitive domains: orientation, memory, attention and calculation, recall, and language, with a total score of 30. A normal MMSE score is defined as ≥ 24 points.

#### Motor score

The motor score primarily assesses the muscle strength of key muscles innervated by different spinal nerves. The total motor score is 100, consisting of an upper extremity motor score and a lower extremity motor score, each contributing 50 points.

### fNIRS

Hemodynamic alterations were monitored using continuous wave fNIRS equipment (Nirsmart, Danyang Huichuang Medical Equipment Co., Ltd., China). The fNIRS electrode cap detected near-infrared light at two wavelengths, 730 and 850 nm. The cap comprised 39 channels, including 30 optodes (15 sources, 15 detectors) spaced at 3 cm between sources and detectors. Detection channels were localized bilaterally in accordance with the 10–20 system. The fNIRS probes and the region of interest (ROI) are shown in Additional file 1: Figure S1AB. During the test, participants were instructed to perform a handgrip task (Additional file 1: Figure S1C).

### Electrophysiological recordings

Given the potential association between fatigue status and M1 excitability, additional analysis was conducted using spTMS and ppTMS.

#### spTMS

spTMS of the M1 facilitated the recording of MEP to assess corticospinal excitability. MEPs were recorded from the abductor pollicis brevis muscle utilizing surface Ag-AgCl electrodes with a diameter of 10 mm. These electrodes were affixed in a belly-tendon montage configuration, and TMS equipment (MEB-9409 C, Japan) equipped with a round coil was employed. The RMT was defined as the minimum stimulus intensity (in % maximum stimulator output) that produces a liminal MEP (over 50 µV in 5 of 10 trials) at rest. spTMS was administered to the hotspot at 80% of the maximum stimulator output, and MEPs were recorded over a 5-second interval.

#### ppTMS

ppTMS (Neurosoft Company, Moscow, Russia) was employed to explore intracortical excitability. A figure-of-eight coil, with its midpoint positioned at the bilateral M1 hand area, was sequentially moved for measurements. TMS stimulation was administered 20 times at 120% RMT stimulation intensity, ensuring each stimulation interval was at least 5 s. The average of the 20 measurements of MEP was used as the baseline MEP. SICI (90% RMT of the first stimulus, 120% RMT of the second stimulus, 2.5 ms interval), ICF (90% RMT of the first stimulus, 120% RMT of the second stimulus, 12 ms interval), and LICI (120% RMT of the first stimulus, 120% RMT of the second stimulus, 200 ms intervals) were subsequently measured. The MEP evoked by the second stimulus (test stimulus) was then compared with the baseline MEP.

### Statistical analysis

#### Descriptive statistics and baseline data comparison

Measurement data were presented as either standard deviation or medians with interquartile ranges. Enumeration data were expressed as number [%] (N [%]). Data in the graphs were reported as mean ± standard error of the mean. The comparison of baseline data utilized the t-test, Chi-squared test, or Fisher’s exact test, depending on the nature of the data distribution (assessed using the Shapiro-Wilk test). Parametric or nonparametric tests were employed accordingly.

In the fifth month of the cohort study, nine patients experienced a reinfection with COVID-19, precluding the acquisition of recovery status at the 6-month disease course. Consequently, our primary analytical approach involved the paired sample test to assess differences between patients at two time intervals (1 m vs. 3 m). The independent sample test was applied to compare differences between patients and healthy controls. Chi-square and McNemar tests were employed for categorical data. A *P-value <* 0.05 was statistically significant.

#### fNIRS data preprocessing and analysis

The fNIRS data preprocessing was conducted using NirSpark software (Danyang Huichuang, China). During the preprocessing stage, we removed irrelevant time intervals and artifacts caused by motion and environmental factors using the automatic motion correction feature. The standard deviation threshold was set to 6.0, and the amplitude threshold was set to 0.5. This software utilizes digital bandpass filtering on the initial optical density signal (0.01–0.2 Hz) to eliminate common sources of noise. Utilizing the modified Beer-Lambert’s law, the intensity counts experimentally measured at the two wavelengths across all channels were converted into relative-change curves of oxygenated hemoglobin (HBO). Given that HBO is more sensitive to cerebral blood flow than deoxyhemoglobin, we chose to use HBO signals as indicators of the hemodynamic response. We computed the average HBO values from the channels covering each ROI. Based on our patient data, HBO levels peak after five seconds of clenching. Therefore, the mean HBO level obtained from 3 to 7 s during each handgrip was used to calculate average HBO values.

#### The balance between hemispheres

To assess interhemispheric balance, the laterality index (LI) was computed using the HBO data from both the DH and non-dominant hemisphere (NDH). The formula for calculating LI is as follows: $LI= \frac{\varvec{\varDelta }HBO\ contralateral-\varvec{\varDelta }HBO\ ipsilateral}{\mid \varvec{\varDelta }HBO\ contralateral\mid +\varvec{\varDelta }\mid HBO\ ipsilateral\mid }$ [15]. Larger LI indicates a greater degree of activation in the contralateral hemisphere. The amplitudes (% of baseline) of ppTMS were used to calculate the hemispheric conversion degree [ln (DH/NDH)], as done in our previous study [16].

#### Correlation analysis and multiple linear regression analysis

To identify independent correlation factors influencing fatigue symptoms following COVID-19 infection, univariate analysis was conducted with FSS scores as the outcome variable. The inclusion criteria for the univariate analysis were set at *P* < 0.1. Variables identified as significant in the univariate analysis were entered into multiple linear regression analysis using the Akaike Information Criterion. Additionally, multivariate regression analysis included age, previously recognized as a significant factor influencing fatigue symptoms [17]. For participants with missing data, telephone follow-up was conducted for primary and secondary outcomes. Data that could not be completed during follow-up were imputed using appropriate means. Statistical analysis was carried out using SPSS 27.0 and R version 4.3.1 for all analyses.

## Results

Table 1 presents the demographic data and basic information for 24 healthy controls and 29 COVID-19 patients. The mean age of the patients was 34.3 ± 6.6 years. No significant differences were observed in age, sex, occupation, and education between the patients and healthy controls (sex, *P =* 0.745; age, *P =* 0.134; education, *P =* 0.681; occupation, *P =* 0.420).

**Table 1:** Participants’ socio-demographic factors, work-related factors

Descriptor	Patient *N* = 29	Control *N* = 24	*P* value
Gender, n (%)^a^			
Female	17(58.6)	13(54.2)	0.745
Male	12(41.4)	11(45.8)	
Age, y, mean (SD)^b^	34.3(6.6)	31.4(7.3)	0.134
Educational attainment, n (%)^c^			
High school or below	1(3.4)	1(4.2)	0.681
Junior college degree	1(3.4)	0(0)	
Bachelor’s degree	19(65.6)	13(54.2)	
Master’s degree	6(20.7)	9(37.5)	
PhD	2(6.9)	1(4.1)	
Occupation, n (%)^c^			
Doctors	8(27.6)	11(47.5)	0.420
Nurses	8(27.6)	3(19.3)	
Therapists	11(37.9)	8(14.4)	
Other healthcare workers	2(6.9)	2(18.8)	

y, year; SD, standard deviation

^a^Chi-square test

^b^Independent two-sample t-test

^c^Fisher’s exact chi-square test

### Fatigue symptoms worsen at 1 m and returned to normal levels at 3 m after infection

**Fig. 2:**
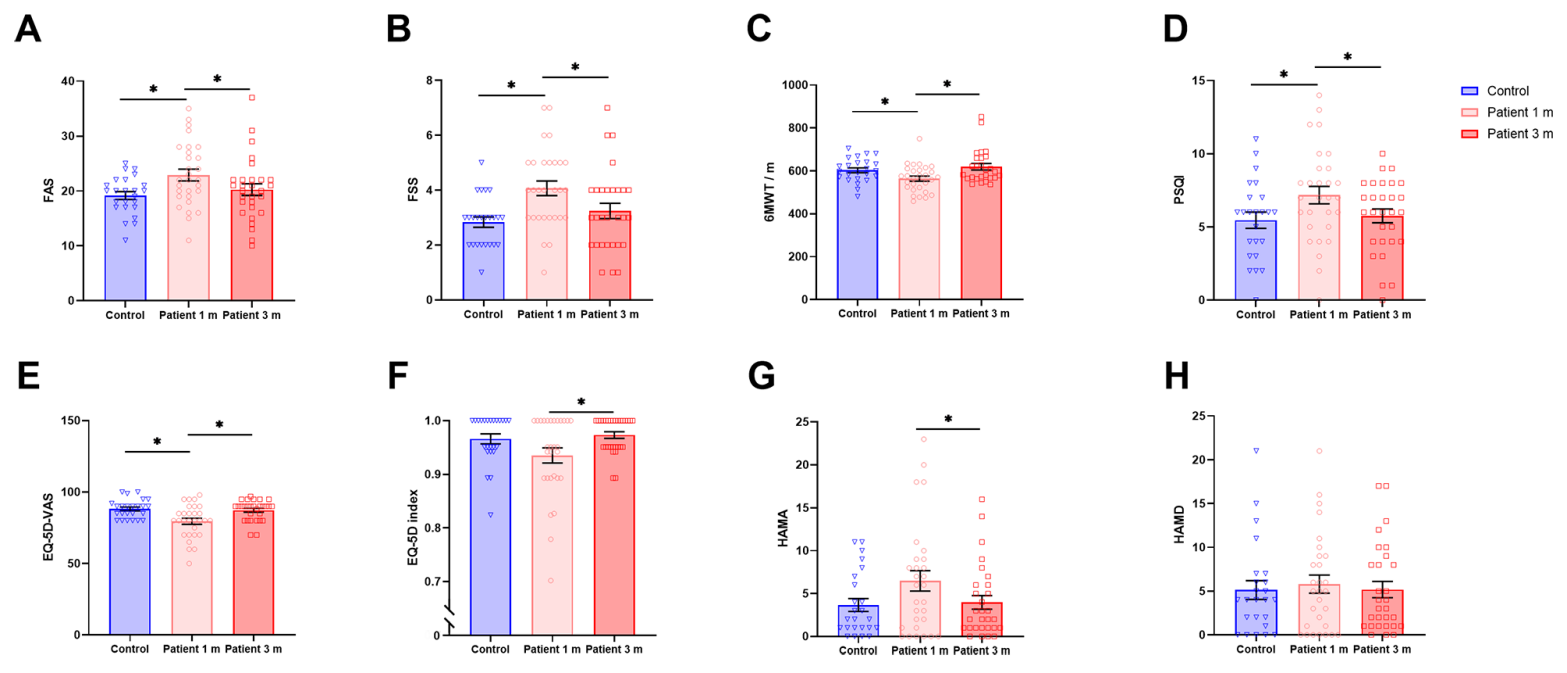
Comparison of fatigue, physical capacity, sleep and health quality, and psychological status between healthy controls and COVID-19 patients at different time points. **A-B** Comparison of fatigue symptoms between COVID-19 patients and healthy controls through FAS and FSS. **C** Comparison of physical capacity between patients and healthy controls through 6MWT. **D** Comparison of sleep quality between patients and healthy controls through PSQI. **E** Comparison of health quality between patients and healthy controls through EQ-5D-VAS. **F** Comparison of health quality between patients and healthy controls through EQ-5D index. **G** Comparison of anxiety between patients and healthy controls through HAMA. **H** Comparison of depression between patients and healthy controls through HAMD. Horizontal black lines above data sets mark significant differences (*P < 0.05, **P < 0.01, ***P < 0.001). Abbreviations: FAS, fatigue assessment scale; FSS, fatigue severity scale; 6MWT, 6-minute walking test; PSQI, pittsburgh sleep quality index; EQ-5D-VAS, euroqol five-dimensional questionnaire visual analogue scale; EQ-5D Index, euroqol health-utility index; HAMA, hamilton anxiety scale; HAMD, hamilton depression scale

At 1 m following COVID-19 infection, 55.2% of healthcare workers exhibited symptoms of fatigue (FAS > 21), with 37.9% experiencing clinically significant levels (FSS ≥ 5). The fatigue degree was significantly higher than that of healthy controls (FAS, *P =* 0.006; FSS, *P* < 0.001; Fig. 2AB, Additional file 1: Table S1). At 3 m, fatigue symptoms markedly improved, with no statistically significant difference between groups (patient 3 m vs. control, FAS, *P =* 0.425; FSS, *P =* 0.251; Fig. 2AB, Additional file 1: Table S1). However, 34.5% of patients still reported self-experienced fatigue, with 10.3% reporting significant fatigue, which was higher than that of the healthy controls (25.0% of healthcare workers reported fatigue symptoms, and 4.2% reported significant fatigue).

Physical capacity results showed that compared to the healthy controls (603 ± 54 m), COVID-19 patients showed a decreased 6MWT walking distance at 1 m (564 ± 62 m, *P =* 0.023; Fig. 2C, Additional file 1: Table S1), and returned to normal levels by 3 m (619 ± 79 m, *P =* 0.397; Fig. 2C, Additional file 1: Table S1).

The PSQI results revealed that over 44.8% of healthcare workers reported poor sleep quality at 1 m after infection, which mainly manifested as longer sleep latency and less sleep time. By 3 m after COVID-19 infection, sleep disturbances significantly eased (*P* = 0.008; Fig. 2D, Additional file 1: Table S1).

The EQ-5D-5L showed that EQ-5D-VAS and EQ-5D index were significantly higher at 3 m than at 1 m (EQ-5D-VAS, *P =* 0.001; EQ-5D index, *P =* 0.024; Fig. 2EF, Additional file 1: Table S1). No significant difference was found in EQ-5D-VAS and EQ-5D index between patients in 3 m and healthy controls (EQ-5D-VAS, *P =* 0.626; EQ-5D index, *P =* 0.516; Fig. 2EF, Additional file 1: Table S1).

The mental health assessment results showed that 44.8% of the COVID-19 patients reported anxiety symptoms (HAMA ≥ 7), and 34.5% reported depressive symptoms (HAMD > 7) at 1 m after infection. At 3 m, the anxiety levels significantly alleviated (*P =* 0.042; Fig. 2G, Additional file 1: Table S1). There was no significant change in depressive mood (*P* > 0.05; Fig. 2H, Additional file 1: Table S1).

None of the patients were estimated as having cognitive impairment according to the MMSE results (Additional file 1: Table S1). Motor scores were normal in all participants (Additional file 1: Table S1).

Consequently, COVID-19 patients exhibited notably deteriorating performances in fatigue, sleep quality, quality of life, and anxiety state compared to healthy controls at 1 m, but recovered at 3 m. No significant change was found in depressed mood.

### Brain activation and excitability changed after COVID-19 infection

#### Activation of bilateral PFC decreased after infection

**Fig. 3:**
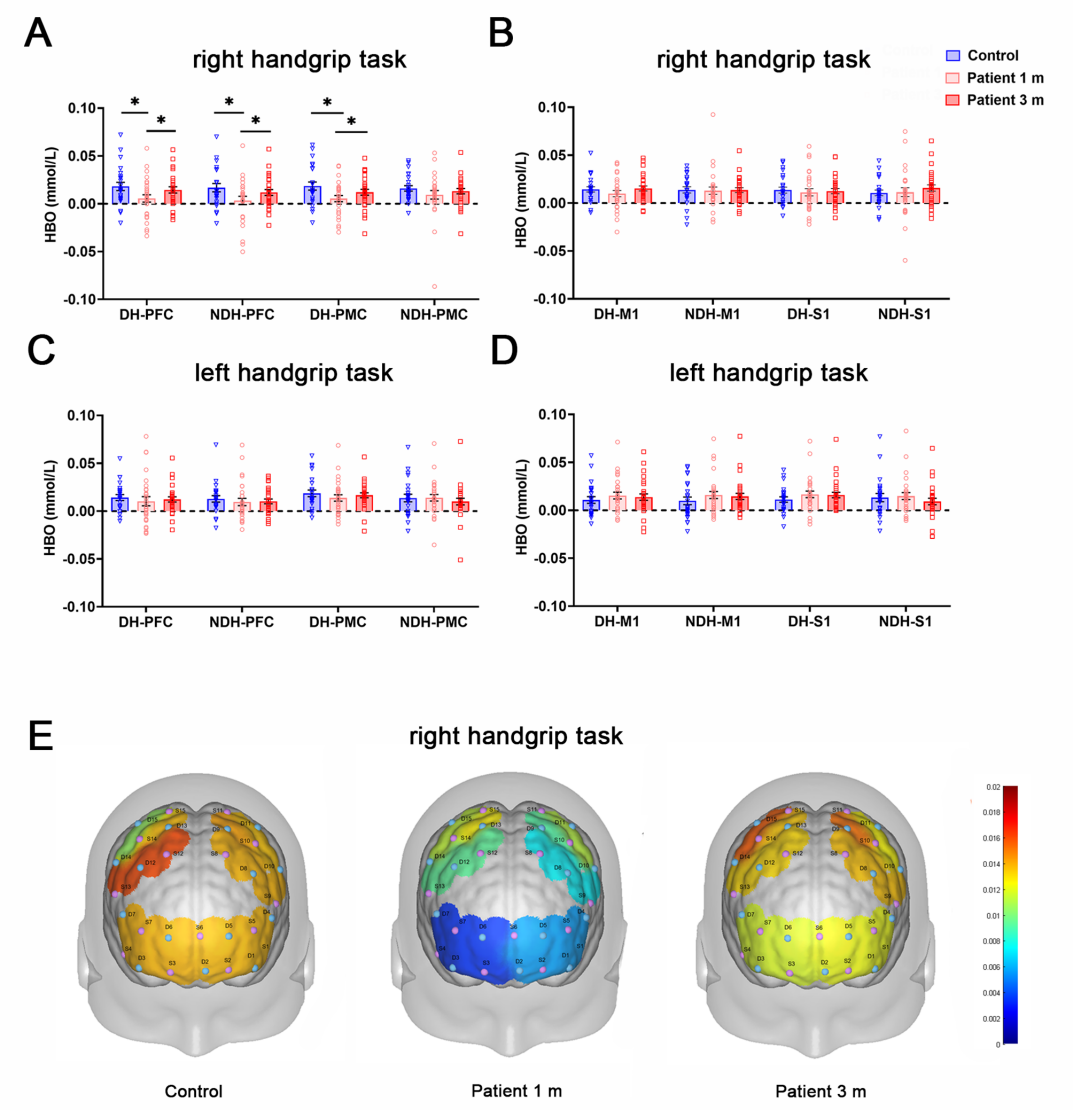
Comparison of brain activation between patients and healthy controls through fNIRS. **A-B** Differences in brain activation (HBO) between the patients and healthy controls based on ROI analysis during the right handgrip task. **C-D** The activation of blood oxygen in each ROI during the left handgrip task. **E** Brain activation during the right handgrip task between the patients and healthy controls based on ROI. Horizontal black lines above data sets mark significant differences (*P < 0.05, **P < 0.01, ***P < 0.001). Abbreviations: fNIRS, functional near-infrared spectroscopy; HBO, oxygenated hemoglobin; ROI, region of interest; PFC, prefrontal cortex; PMC, premotor cortex; M1, primary motor cortex; S1, primary somatosensory area; DH, dominant hemisphere; NDH, non-dominant hemisphere

To investigate alterations in cerebral cortex activity after COVID-19 infection, fNIRS was employed to examine cerebral hemodynamics. At 1-month post-infection, COVID-19 patients displayed a general decline in cortex activation compared to healthy controls. During the right handgrip task, activation of bilateral PFC (DH-PFC: 0.006 ± 0.021 mmol/L, NDH-PFC: 0.003 ± 0.024 mmol/L) and DH-PMC (0.006 ± 0.018 mmol/L) was significantly lower than that of healthy controls (*P* < 0.05, Fig. 3A). Bilateral M1 also showed a downward trend in brain activation at 1 m (*P* > 0.05, Fig. 3B). However, at 3 m, HBO levels in bilateral PFC and DH-PMC gradually returned to levels comparable to healthy controls (*P* > 0.05, Fig. 3A). No significant change was observed in activation between patients and healthy controls during the left handgrip task (*P* > 0.05, Fig. 3CD). Figure 3E illustrates alterations in brain activation regions during the right handgrip task between patient groups and healthy controls at different time points.

#### Bilateral M1 area excitability decreased with more decrease in DH side after infection

**Fig. 4:**
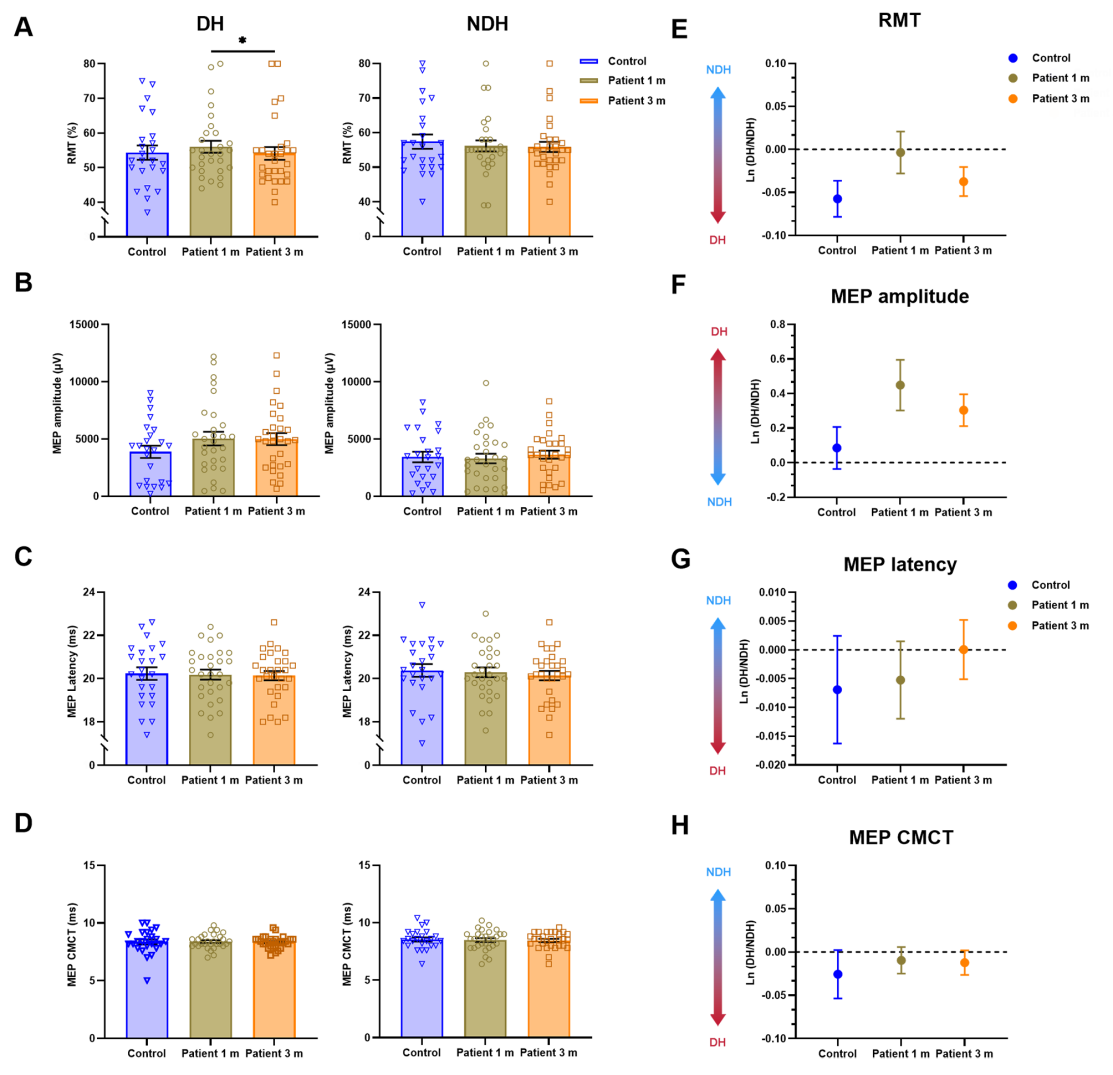
Comparison of M1 area RMT and MEP of patients and healthy controls. **A** Comparison of M1 hand area RMT of patients and healthy controls. **B-D** Comparison of M1 hand area MEP amplitude, MEP latency, and MEP CMCT between patients and healthy controls. **E-H** The balance between hemispheres was calculated as the ln(DH/NDH) of M1 hand area RMT, MEP amplitude, MEP latency, and MEP CMCT. The more red the ln(DH/NDH), the more dominant the DH. Horizontal black lines above data sets mark significant differences (*P < 0.05, **P < 0.01, ***P < 0.001). Abbreviations: M1, primary motor cortex; MEP, motor evoked potential; RMT, resting motor threshold; DH, dominant hemisphere; NDH, non-dominant hemisphere; CMCT, central motor conduction time

The intensity of the DH RMT in patients at 1 m after COVID-19 infection was 56.0 ± 9.3, decreasing to 54.1 ± 10.3 at 3 m with a significant difference (*P* < 0.05, Fig. 4A). The RMT of the DH in the healthy control group was 54.3 ± 10.1, showing no significant difference compared to the patient group. There was no statistically significant distinction in RMT comparisons of the NDH between groups. MEP was measured to further investigate alterations in corticospinal excitability after COVID-19. No significant differences were observed in MEP amplitude (Fig. 4B), latency (Fig. 4C), or central motor conduction time (CMCT, Fig. 4D) between healthy controls and patients. The hemispheric conversion degree [ln(DH/NDH)] was subsequently calculated to observe bilateral hemispheric differences and functional changes. Despite the absence of any significant statistical differences between the groups (Fig. 4E-H), the RMT-ln(DH/NDH) and amplitude-ln(DH/NDH) of MEP exhibited an upward trend at 1 m when compared to the healthy control group (*P*>0.05, Fig. 4EF).

**Fig. 5:**
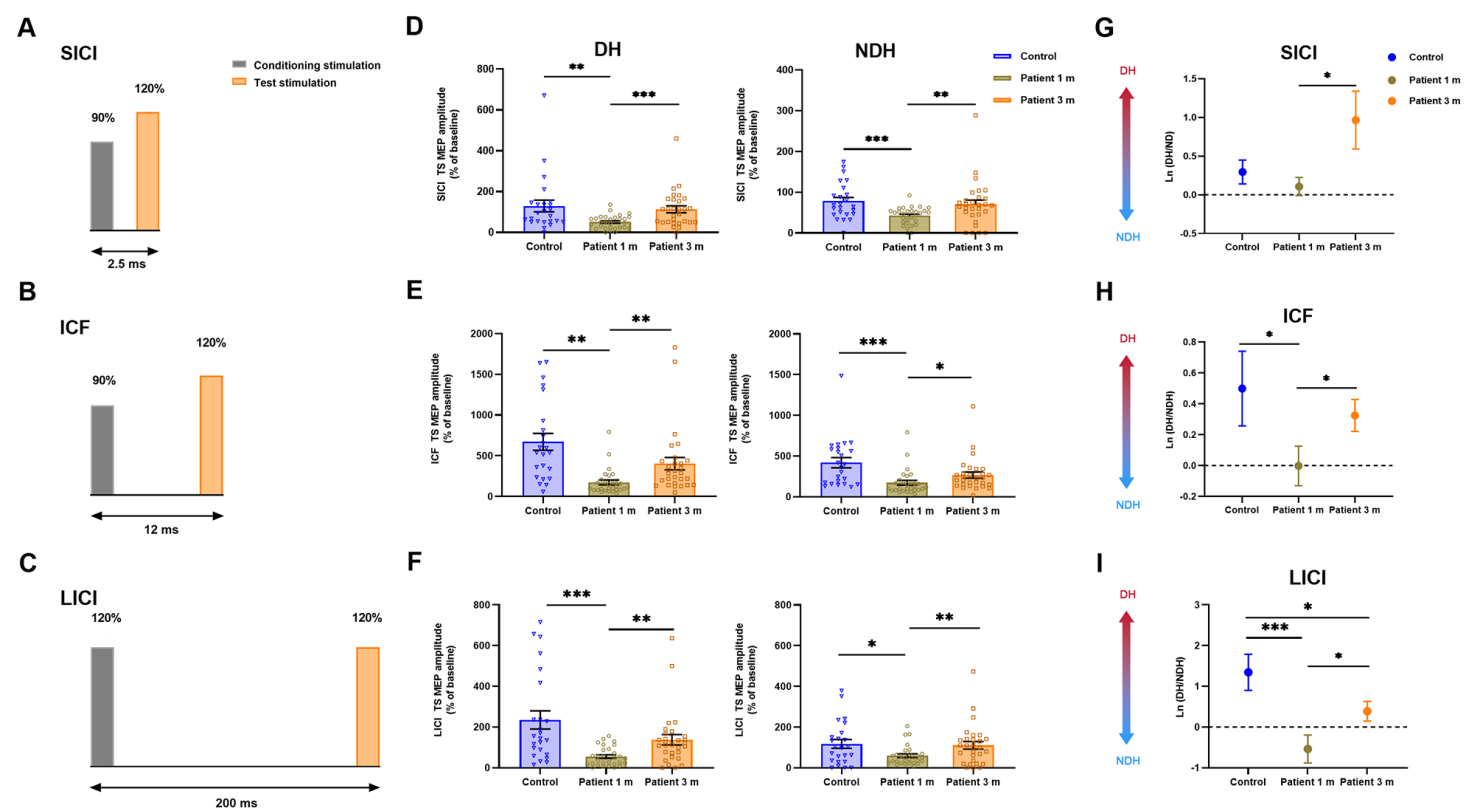
Comparison of M1 area amplitudes (% of baseline) of ppTMS between patients and healthy controls. **A-C** The stimulation interval of ppTMS. **D-F** Comparison of M1 hand area amplitudes (% of baseline) of ppTMS between patients and healthy controls in SICI, ICF, and LICI. **G-H** Comparison of the M1 hand area ln(DH/NDH) of amplitudes (% of baseline) of ppTMS ln(DH/NDH) between patients and healthy controls. The greater the ln(DH/NDH), the more red it is, the greater the dominance of the dominant hemisphere. Horizontal black lines above data sets mark significant differences (*P < 0.05, **P < 0.01, ***P < 0.001). Abbreviations: M1, primary motor cortex; ppTMS, paired pulse TMS SICI, short interval intracortical inhibition; ICF, intracortical facilitation; LICI, long-interval intracortical inhibition; DH, dominant hemisphere; NDH, non-dominant hemisphere

To further investigate changes in intracortical excitability, we conducted ppTMS measurements. Figure 5A-C illustrates the stimulation interval paradigm of ppTMS. Compared to the healthy control group, SICI, ICF, and LICI in the bilateral M1 area significantly decreased following COVID-19 infection at 1 m (*P* < 0.05, Fig. 5D-F). At 3 m, bilateral SICI, ICF, and LICI substantially recovered to levels comparable to those in the healthy controls (*P*>0.05, Fig. 5D-F). Upon calculating the hemispheric conversion degree [ln(DH/NDH)], our results indicated that SICI-ln(DH/NDH) in patients decreased at 1 m (0.107 ± 0.6). At 3 m, the SICI-ln(DH/NDH) increased to 0.965 ± 2.0 compared to 1 m (*P =* 0.027, Fig. 5G). No significant difference was found between the patient group and the healthy control group (*P*>0.05, Fig. 5G). Similarly, the patients’ ICF-ln(DH/NDH) at 1 m was − 0.002 ± 0.7 and significantly increased to 0.318 ± 0.6 at 3 m (*P =* 0.024, Fig. 5H). The ICF-ln(DH/NDH) of the healthy control group was 0.499 ± 1.2, which was significantly higher than that at 1 m (*P* = 0.033, Fig. 5H). The LICI-ln(DH/NDH) was − 0.537 ± 1.8 at 1 m, and increased to 0.402 ± 1.3 at 3 m (*P* = 0.041, Fig. 5I). The LICI-ln(DH/NDH) of the healthy control group was 1.346 ± 2.2, which was significantly higher than that at 3 m (*P* = 0.036, Fig. 5I).

In summary, our findings suggest a significant decrease in bilateral M1 excitability after COVID-19 infection, and the hemispheric conversion degree further identifies a more pronounced decrease in excitability on the DH side.

### The activation of PFC and the excitability of the M1 region are strongly associated with fatigue

In light of the substantial changes observed in brain regions’ activation and excitability in COVID-19 patients at 1 m after infection, we conducted a detailed analysis to explore the correlation between these brain alterations and fatigue symptoms. The FSS score at 1 m was selected as the outcome measure, and cerebral blood oxygen concentration along with electrophysiological recordings at 1 m were utilized as independent variables.

#### The lower the activation of the bilateral PFC, the more severe the fatigue symptoms

At 1 m after COVID-19 infection, the cross-sectional analysis revealed that NDH-PFC (unstandardized β=-30.612, *P* = 0.008; Table 2), PFC-LI (β=-1.243, *P* = 0.041; Table 2), M1-LI (unstandardized β = 1.070, *P* = 0.017; Table 2), and age (unstandardized β=-0.072, *P* = 0.037; Table 2) were independent factors contributing to fatigue. The adjusted R^2^ = 0.440. The correlation analysis results for NDH-PFC suggested that lower NDH-PFC activation was associated with increased fatigue (NDH-PFC, *r*=-0.456, *P* = 0.013; Additional file 1: Figure S2A). Similarly, PFC-LI results indicated that lower DH-PFC activation compared with NDH-PFC was linked to higher levels of fatigue (PFC-LI, *r*=-0.461, *P* = 0.012; Additional file 1: Figure S2B). Interestingly, both PFC-LI and M1-LI exhibited a declining trend at 1 m of disease and recovered at 3 m compared with healthy controls (*P* > 0.05, Additional file 1: Figure S2C-D). Regarding age, younger patients experienced a higher degree of fatigue (unstandardized β=-0.072, *P* = 0.037; Table 2).

**Table 2:** Multiple linear regression analysis at 1 m

Variable	Patient 1 m	Univariate analysis (1 m FSS)			Multivariate analysis (1 m FSS)	
		Unstandardized β (95% CI)	*P *value		Unstandardized β (95% CI)	*P *value
DH-PFC^#^	0.010 ± 0.026	-0.443 (-0.696, -0.091)	**0.016**			
NDH-PFC^#^	0.010 ± 0.021	-0.456 (-0.705, -0.107)	**0.013**		-30.612 (-52.256, -8.969)	**0.008**
DH-PMC^#^	0.014 ± 0.018	-0.374 (-0.651, -0.009)	**0.046**			
DH-PFC^※^	0.006 ± 0.021	-0.359 (-0.641, 0.009)	0.056			
NDH-S1^#^	0.015 ± 0.020	-0.350 (-0.635, 0.019)	0.063			
PFC-LI^※^	-0.029 ± 0.384	-0.461 (-0.708, -0.114)	**0.012**		-1.243 (-2.431, -0.054)	**0.041**
M1-LI^※^	-0.154 ± 0.522	0.312 (-0.061, 0.609)	0.099		1.070 (0.205, 1.936)	**0.017**
NDH-SICI	45.009 ± 21.226	0.404 (0.043, 0.671)	**0.03**			
SICI-ln(DH/NDH)	0.107 ± 0.636	-0.426 (-0.686, -0.071)	**0.021**			
Age	34.280 ± 6.579	-0.138 (-0.480, 0.241)	0.475		-0.072 (-0.139, -0.005)	**0.037**

FSS, fatigue Severity Scale; DH, dominant hemisphere; NDH, non-dominant hemisphere; PFC, prefrontal cortex; PMC, premotor cortices; S1, primary somatosensory area; LI, laterality index; SICI, short interval intracortical inhibition

Significant *P* values are highlighted in bold

^**#**^during left handgrip task

^**※**^during right handgrip task

Adjusted R^2^ = 0.440

#### The greater the excitability recovery in the M1 region, the more significant the improvement in patient fatigue

At 3 m, patients’ fatigue symptoms had largely returned to healthy control levels. However, 10.3% of the patients still reported severe fatigue. Understanding the factors influencing post-COVID-19 fatigue is crucial for enhancing patient recovery and disease management. Therefore, we conducted further analysis to explore the relationship between changes in brain function indicators and changes in fatigue from 1 to 3 m after COVID-19 infection.

The results of multiple regression revealed that the variation in DH-LICI (unstandardized β=-0.005, *P* = 0.009; Table 3) and NDH-latency (unstandardized β = 0.639, *P* = 0.015; Table 3) were independent factors influencing the improvement in fatigue, with an adjusted R^2^ = 0.264. Specifically, the greater the increase in DH-LICI, the more significant the improvement in fatigue (*r*=-0.374, *P* = 0.046; Additional file 1: Figure S3A). Additionally, a shorter NDH-latency of MEP was associated with greater fatigue improvement (*r* = 0.332, *P* = 0.079; Additional file 1: Figure S3B). These findings suggest that the enhancement of excitability in bilateral hemispheres significantly contributes to the improvement of fatigue.

**Table 3:** Multiple linear regression analysis

Variable (△=3 m–1 m)	Univariate analysis (△FSS = 3 m–1 m)			Multivariate analysis (△FSS = 3 m–1 m)	
	Unstandardized β (95% CI)	*P *value		Unstandardized β (95% CI)	*P *value
NDH-M1^※^	-0.327 (-0.619, 0.045)	0.084			
NDH-ICF	-0.324 (-0.617, 0.048)	0.086			
DH-LICI	-0.374 (-0.651, -0.008)	**0.046**		-0.005 (-0.009, -0.001)	**0.009**
NDH-Latency	0.332 (-0.039, 0.623)	0.079		0.639 (0.133, 1.145)	**0.015**
DH-Latency	0.351 (-0.018, 0.636)	0.062			
NDH-CMCT	0.316 (-0.057, 0.611)	0.095			
Age	-0.138 (-0.480, 0.241)	0.475			

FSS, fatigue severity scale; DH, dominant hemisphere; NDH, non-dominant hemisphere; M1, primary motor cortex; ICF, intracortical facilitation; LI, laterality index; LICI, long-interval intracortical inhibition; CMCT, central motor conduction time

Significant *P* values are highlighted in bold

^**※**^during right handgrip task

Adjusted R^2^ = 0.264

### Fatigue symptoms and brain excitability/activation tended to worsen in re-infected COVID-19 patients

At 6 m, all fatigue symptoms and cortical excitability were assessed. Due to 9 patients experiencing a reinfection with COVID-19, the data of COVID-19 patients at 6 m were categorized into two groups: the COVID-19 single-infected group (*N* = 20) and the COVID-19 re-infected group (*N* = 9). Additional file 1: Table S2 presents the basic information. In comparison with the single-infected group, the re-infected group exhibited a renewed increase in fatigue scores at 6 m (*P* > 0.05, Additional file 1: Figure S4A). Additionally, the activation of the PFC and the excitability of the M1 showed a downward trend (*P* > 0.05, Additional file 1: Figure S4B-F).

Overall, there was a significant trend in cortical activation/excitability among re-infected patients at 6 m post-initial COVID-19 infection.

## Discussion

**Fig. 6:**
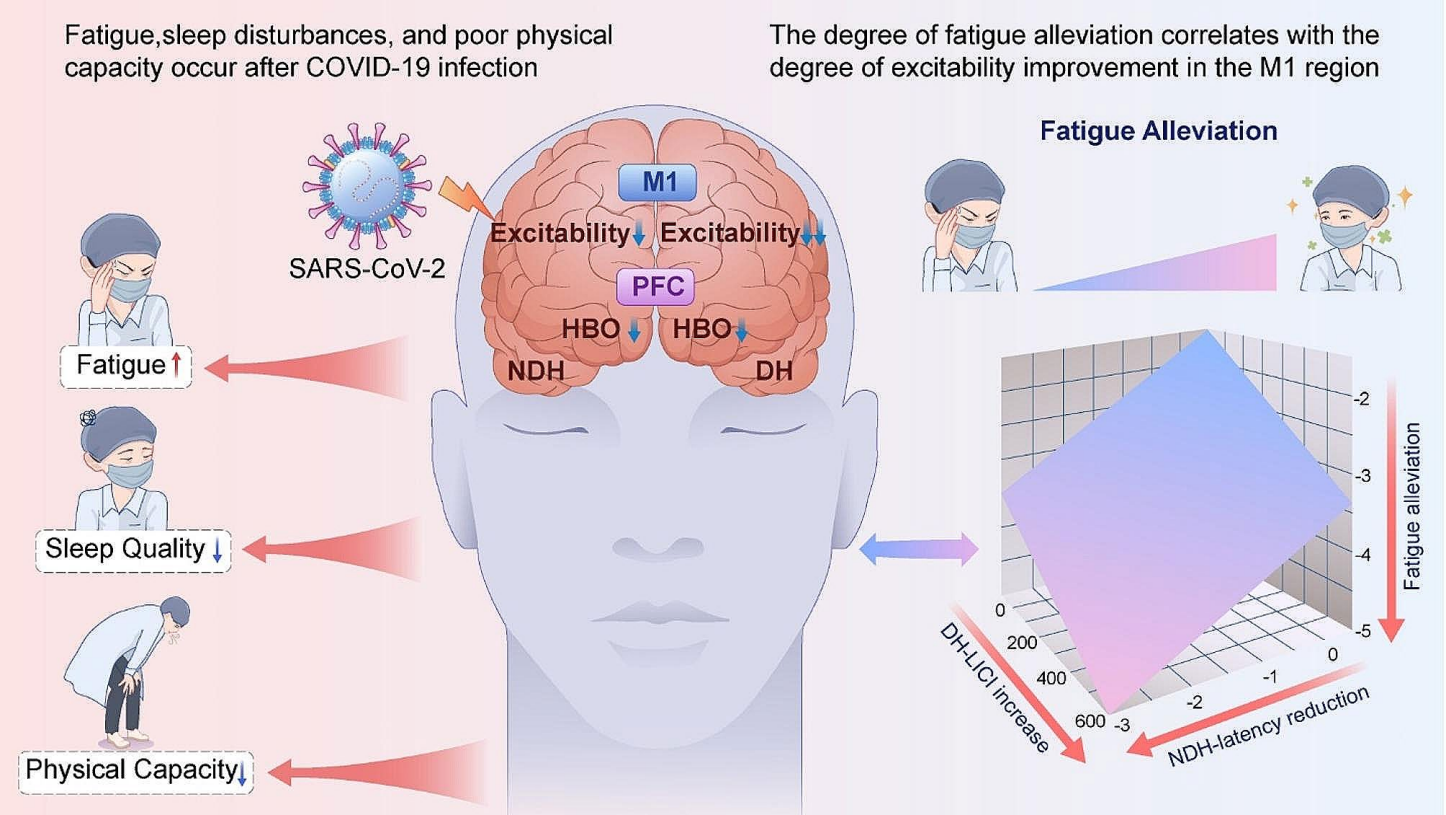
Central mechanisms of post-COVID-19 fatigue. Healthcare workers suffered from fatigue, sleep disorders, and poor physical capacity after COVID-19 infection. The cerebral cortex exhibited reduced activation and excitability in the PFC and M1 at 1 m. Multiple linear regression analysis revealed that the increase in DH-LICI and the decrease in NDH-latency were associated with the more significant improvement in fatigue symptoms. Abbreviations: PFC, prefrontal cortex; M1, primary motor cortex; LICI, long-interval intracortical inhibition; HBO, oxygenated hemoglobin; DH, dominant hemisphere; NDH, non-dominant hemisphere

Our study indicates that COVID-19 infection leads to fatigue in healthcare workers. At 1 m after infection, 37.9% of healthcare workers reported significant fatigue, which decreased to 10.3% at 3 m, approaching the levels observed in healthy controls. The cerebral cortex exhibited reduced activation and excitability in the PFC and M1 at 1 m. Multiple linear regression analysis revealed that greater changes in M1 excitability were associated with more significant improvement in fatigue symptoms (Fig. 6). Moreover, COVID-19 reinfection led to a renewed decrease in M1 excitability at 6 m.

### COVID-19 leads to long-term fatigue

Currently, healthcare workers continue to face a heightened risk of COVID-19 infection. Prolonged fatigue has been commonly reported following COVID-19 infection. Our findings indicate that fatigue symptoms among healthcare workers improved at 3 m post-infection, a shorter duration than the reported 6-month fatigue in the study by Huang C et al. [18]. This discrepancy may be attributed to the fact that all participants in our study had mild COVID-19, whereas the study by Huang C et al. included hospitalized patients with more severe symptoms. Additionally, the evolution of the novel coronavirus may contribute to symptom relief after infection [19]. Long-term fatigue symptoms post-COVID-19 may be associated with sleep quality. Our results revealed that 44.8% of patients experienced sleep disorders at 1 m after COVID-19 infection, characterized by prolonged sleep latency and shortened sleep duration. Medical staff, often subjected to night shifts, may experience discomfort from COVID-19 infection, contributing to poor sleep quality. Age also emerged as a significant factor in fatigue symptoms, with our findings indicating that younger patients experienced more severe fatigue symptoms, contrary to the traditional belief that older individuals would be more fatigued. Indeed, it should be borne in mind that our study population, primarily aged 25–35 years, represents the core demographic of the hospital staff, with younger medical personnel being more directly involved in frontline COVID-19 treatment.

Fatigue can be categorized into central fatigue and peripheral fatigue. Our study ruled out peripheral fatigue due to normal levels of key muscle strength in all patients. In contrast, the pathogenesis of central fatigue has broader physiological implications and is more challenging to recover from. Even in cases of mild COVID-19, persistent inflammatory responses have been observed in brain tissues several months after infection [20]. These findings suggest that long-term fatigue may be linked to the enduring effects of COVID-19 on the brain. Consequently, we sought to explore the relationship between fatigue symptoms and cortical changes following COVID-19 infection.

### Decreased activation and excitability of PFC/M1 are correlated with fatigue symptoms after COVID-19

#### PFC

The PFC plays a crucial role in the occurrence of fatigue. Previous research has indicated that COVID-19 can disrupt the blood-brain barrier, resulting in inflammatory changes such as neurophagocytosis in the PFC [21]. Consequently, we initiated our investigation by assessing the hemodynamic function of the PFC using fNIRS. According to the neurovascular coupling theory, increased neural activity triggers local blood flow changes [22]. Our study revealed significantly lower PFC activation in COVID-19 patients compared to healthy controls during a right handgrip task, consistent with findings from a previous magnetic resonance imaging (MRI) study. MRI evidence confirmed that SARS-CoV-2 could preferentially and directly target the PFC area, likely via olfactory epithelium retrograde axonal transport [23], suggesting that the reduction in PFC activation may be linked to the direct invasion of SARS-CoV-2. Cross-sectional analysis indicated that reduced activation in the bilateral PFC was associated with more severe fatigue symptoms. In line with these findings, Caseras X et al. [24] reported reduced dorsolateral and dorsomedial PFC activation in patients with fatigue syndrome. Thus, it is highly conceivable that the invasion of the PFC by the novel coronavirus may exacerbate fatigue symptoms. Furthermore, within the PFC, pathways connecting different cortical regions can enhance signals for cognitive operations that regulate anxiety processing [25]. This may account for the observed increase in anxiety among COVID-19 patients compared to healthy controls.

#### M1

Alongside PFC, the laterality index of M1 (M1-LI) emerged as an independent correlation factor with fatigue. The M1 plays a crucial role in controlling and executing voluntary movements, serving as the primary site for generating nerve impulses during movement [26]. When M1 excitability decreases, the neural drive to descending spinal motor neurons also decreases, resulting in reduced motor motivation and increased fatigue. fNIRS results indicated a downward trend in the activation of the bilateral M1 region and M1-LI in COVID-19 patients at 1 m after infection. The more bilateral M1 activation decreased, the more severe the fatigue experienced by patients. This finding is consistent with a study by Li et al. [27], which showed a decrease in oxygenated hemoglobin in the PFC and M1 after exercise in a fatigue condition, suggesting a decrease in neuronal activity.

SICI and LICI are mediated by GABA_A_ and GABA_B_ receptors, respectively, while ICF is mediated by NMDA receptors. The decrease in SICI, LICI, and ICF collectively reflects the enhancement of GABA receptor-mediated activity and the reduction of NMDA receptor-mediated activity in the M1 at 1 m after COVID-19 infection. These changes are closely related to fatigue and align with the central fatigue model proposed in previous studies. Firstly, the heightened intracortical inhibitory activity in the M1 region results in a diminished neural drive from the motor cortex to the downstream spinal cord motor neurons [28]. Consequently, patients manifest a decrease in energy, reduced activity, and an overall sense of fatigue. This was corroborated by observed lower walking distances in the 6MWT at 1 m post-COVID-19 infection. Secondly, during fatigue, the response of spinal motor neurons to the descending output from the M1 region weakens, leading to a reduction in firing rate. This necessitates additional descending cortical drive to sustain the activation of spinal motor neurons and muscle strength. MEP, indicative of corticospinal excitability, revealed an increase in the amplitude of DH MEP at 1 m. Consequently, it is highly likely that this phenomenon accounts for the compensatory mechanism between the peripheral and central systems. Ortelli P et al. [9] observed that the cortical silent period was prolonged in fatigue patients post-COVID-19 infection compared to healthy controls, further suggesting altered M1 GABAergic neurotransmission in post-COVID-19 fatigue.

Additionally, the diminished ICF implies a reduction in NMDA receptor-mediated activity post-COVID-19 infection. Both ICF results and elevated RMT indicate a significant decrease in bilateral M1 excitability following COVID-19 infection. The analysis of ICF-ln(DH/NDH) further reveals a more pronounced decrease on the DH side. Our spTMS and ppTMS results align with the literature. Ortelli P et al. observed higher RMT in COVID-19 patients, signifying reduced excitability in the dominant M1 area [9]. Our research not only supports this perspective but also uncovers that the degree of fatigue in patients after COVID-19 infection is related not only to the excitability of the dominant M1 but also to the non-dominant M1, suggesting potential intervention targets for improving fatigue symptoms. In conclusion, TMS results suggest that fatigue after COVID-19 infection may be linked to enhanced inhibitory receptor-mediated activity and decreased excitatory receptor-mediated activity, indicating a disruption in the inhibitory/excitatory status of M1 after COVID-19 infection.

### Regulation of inhibitory/excitatory status of M1 might be the basis for further intervention for fatigue

To comprehensively understand the major factors impacting post-COVID-19 fatigue for facilitating recovery and alleviating fatigue symptoms, we conducted a thorough analysis of the correlation between alterations in brain function and fatigue levels occurring at 1 to 3 months after COVID-19 infection. Multiple regression results indicated that the change in DH-LICI in M1 and NDH-latency of MEP are independent influencing factors of the change in FSS score. As mentioned earlier, increased LICI in patients suggests a lower level of inhibition in the M1 compared to healthy controls. A shorter MEP latency implies faster nerve conduction from the center to the periphery. Our results demonstrated that the greater increase in DH-LICI and/or reduction in NDH latency in MEP correspond to a more significant improvement in fatigue symptoms. This aligns with previous studies indicating that the cortical inhibitory system is enhanced and the excitatory system decreases after fatigue [29]. Therefore, the regulation of the inhibitory/excitatory status of the M1 area strongly suggests a potential role for targeted fatigue treatment. It is well-established that high-frequency rTMS (≥ 5 Hz) can improve neuron excitability, while low-frequency rTMS (≤ 1 Hz) inhibits neuron excitability [30]. Fierro B et al. [31] observed a significant increase in LICI, reflecting a reduction of cortical inhibition after 10 Hz rTMS treatment in patients with Parkinson’s Disease. Sasaki N et al. [32] used ten sessions of 10 Hz rTMS targeting the bilateral dorsolateral PFC and occipital lobe in COVID-19 patients with chronic fatigue and cognitive impairment. The results showed a significant alleviation of fatigue symptoms and improvement in intelligence scores, accompanied by enhanced blood perfusion in the PFC and occipital lobes. Drawing from this treatment strategy, targeted intervention to regulate and restore the excitability of bilateral M1 to a normal status might improve fatigue symptoms in COVID-19 patients at the early stage, thereby accelerating rehabilitation. Presently, post-COVID-19 fatigue primarily focuses on self-rest and adjustment, lacking targeted measures for alleviating long-term fatigue symptoms. Our study demonstrated alterations in cortical excitability in patients experiencing post-COVID-19 fatigue. Importantly, the observed cortical changes were not exclusive to post-COVID-19 fatigue but were also present in chronic fatigue syndrome and multiple sclerosis fatigue. Consequently, our study provides additional neuroelectrophysiological evidence of fatigue-related cortical changes, contributing to enhanced clinical treatment approaches for accelerating fatigue relief. Future research could also further explore the effects of manipulating GABA_B_ receptor activity on the improvement of fatigue in animal experiments.

### Reinfection with COVID-19 can once again lead to fatigue recurrence and changes in cortical excitability

As the world transitions into the post-COVID-19 period, medical workers continue to face an elevated risk of COVID-19 reinfection. Our research, encompassing nearly 31% of healthcare workers, revealed instances of SARS-CoV-2 re-infection within a 5-month disease course. A recent *Lancet* study reported that multiple or persistent SARS-CoV-2 infections can lead to cognitive impairment, with ongoing effects on brain function felt up to two years after the initial infection [33]. Our fNIRS and TMS results confirmed a renewed decrease in brain activation/excitability in patients re-infected with COVID-19 at 6 m, slightly higher than at 1 m but lower than at 3 m. Similar trends were observed in FSS assessments. This underscores the importance of vigilance regarding SARS-CoV-2 re-infection and suggests that monitoring the excitability of the M1 region may aid in managing and instructing the early intervention for COVID-19 fatigue symptoms.

Indeed, our research has certain limitations that should be acknowledged. First, this study only included healthcare workers with mild COVID-19, so the findings may not be directly applied to the general population, the other types of COVID-19, or pneumonia caused by other viruses. The study focused on healthcare workers with mild COVID-19 because that’s the most prevalent type [34]. These individuals often continue working instead of being hospitalized. Therefore, research and targeted early intervention for this population have greater clinical meanings. Second, this study did not classify the virus strains, but only enrolled patients with mild COVID-19 infections based on their clinical phenotypes. Whether severe or critical COVID-19 type has a greater impact on PFC/M1 is still unclear and needs further exploration. Third, this was a single-center prospective and small sample study. In the future, multi-center collaborative research should be conducted to examine the central mechanism of fatigue after COVID-19 infection and to develop predictive models for fatigue. Additionally, it is important to observe the efficacy of interventions such as rTMS on fatigue symptoms in different populations after COVID-19 or other similar virus infections.

By using fNIRS, spTMS, and ppTMS, our study provided hitherto undocumented changes in the cerebral cortex of medical staff after COVID-19 infection and correlated them with fatigue. The research revealed bilateral decreases in brain activation/excitability after COVID-19, particularly on the DH side. At 1 m post-COVID-19 infection, 37.9% of patients experienced severe fatigue symptoms, dropping to 10.3% at 3 m. Long-term fatigue symptoms after COVID-19 may be associated with lower activation of the PFC and decreased excitability of the M1 region. Notably, greater changes in M1 region excitability correlated with more significant fatigue improvement. fNIRS can provide high spatial resolution mapping of brain activity, reflecting the degree of blood oxygen activation in brain regions. TMS can measure neural excitability. According to the neurovascular coupling principle, cerebral blood flow is closely related to neural activity. Therefore, the information of brain activity detected by fNIRS combined with TMS is complementary and mutually confirmed, which makes the conclusion of this study more rigorous and reliable. These pathological changes suggest that targeted interventions to regulate M1 excitability could be a novel strategy for COVID-19 patient rehabilitation. For example, TMS is a noninvasive brain stimulation technique that can modulate cortical excitability. Bilateral high frequency stimulation of the M1 region in patients with post-COVID-19 fatigue may accelerate the recovery of fatigue symptoms. Therefore, our study provides a potential basis for noninvasive brain stimulation to treat post-COVID-19 fatigue. Our study once again highlights the fatigue symptoms experienced by healthcare workers after COVID-19 infection, aligning with previous research findings. Given the unique nature of healthcare work, policymakers should promote more effective treatments for post-COVID fatigue and provide more support and care for healthcare workers who may have difficulty recovering from post-COVID-19 fatigue.

### Electronic supplementary material

Below is the link to the electronic supplementary material.

Additional file 1

## Data Availability

The datasets analyses during the current study are available from the corresponding author on reasonable request.

## Abbreviations

COVID-19: Coronavirus disease 2019
6MWT: 6-minute walking test
RMT: Resting motor threshold
MEP: Motor evoked potential
M1: Primary motor cortex
SICI: Short interval intracortical inhibition
ICF: Intracortical facilitation
LICI: Long-interval intracortical inhibition
fNIRS: Functional near-infrared spectroscopy
PFC: Prefrontal cortex
PMC: Premotor cortex
S1: Primary somatosensory area
NDH: Non-dominant hemisphere
DH: Dominant hemisphere
rTMS: Repeated transcranial magnetic stimulation
FAS: Fatigue assessment scale
FSS: Fatigue severity scale
PSQI: Pittsburgh sleep quality index
EQ-5D-5L: European quality of life five-dimension five-level scale
EQ-5D: index EuroQoL health-utility index
VAS: Visual analogue scale
HAMA: Hamilton anxiety scale
HAMD: Hamilton depression scale
HBO: Oxygenated hemoglobin
ROI: Region of interest
spTMS: Single-pulse TMS
ppTMS: Paired pulse TMS
LI: Laterality index
CMCT: Central motor conduction time
MRI: Magnetic resonance imaging
