# Supplementary material for: PFC/M1 activation and excitability: a longitudinal cohort study on fatigue symptoms in healthcare workers post-COVID-19
Source: J Transl Med. 2024 Aug 5;22:720. doi: 10.1186/s12967-024-05319-z (PMC11299412; doi:10.1186/s12967-024-05319-z)
Supplement: Supplementary file 1 — Additional file 1 [file 12967_2024_5319_MOESM1_ESM.docx]

## **Supplementary Material**

**Figure S1** Layout of the fNIRS probes and channels and experimental paradigm


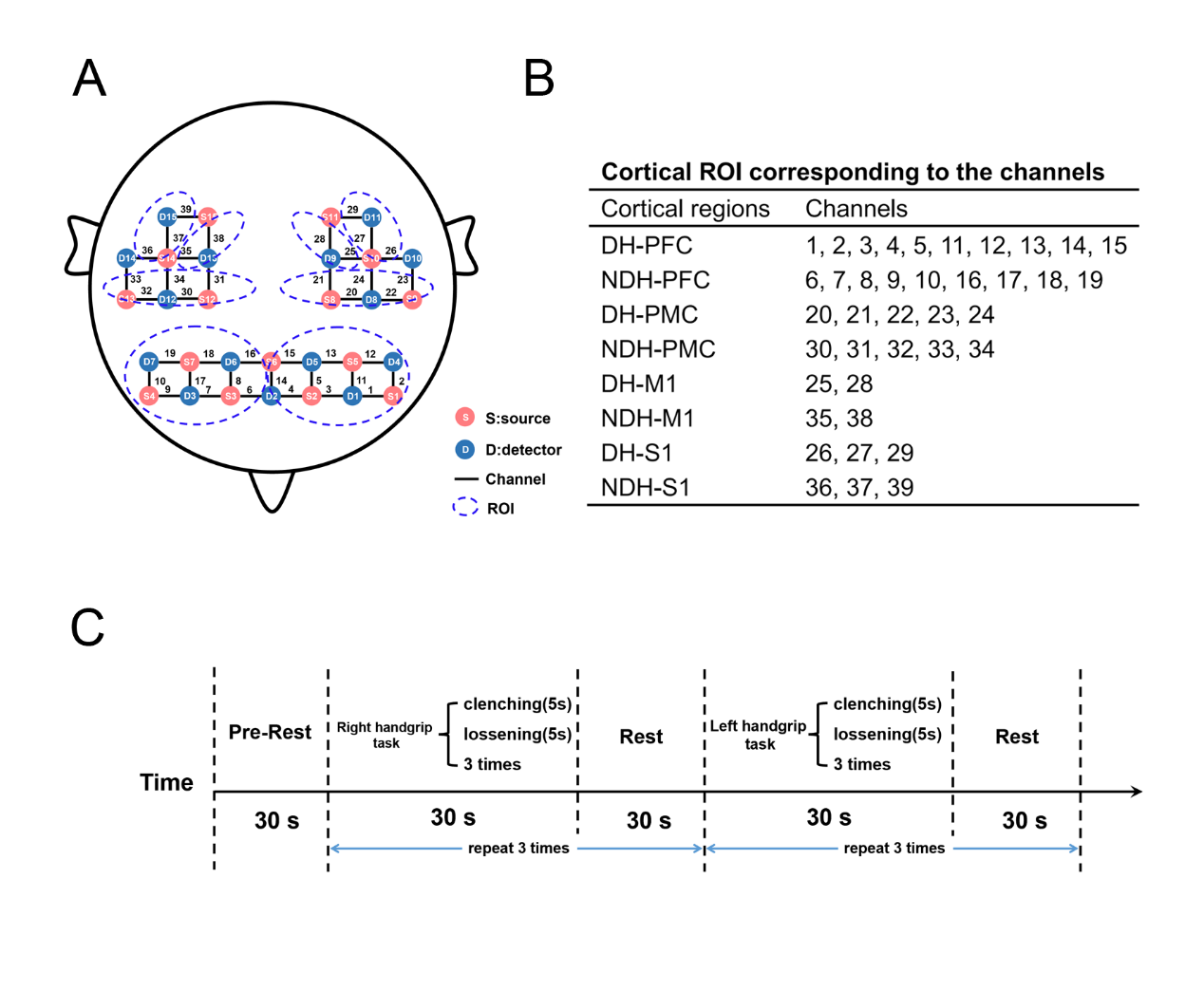


**A** Layout of the fNIRS probes and channels. The 15 pink circles correspond to fNIRS light emitters, while the 15 blue circles correspond to fNIRS detectors. There was a total of 39 fNIRS channels. **B** Cortical ROI corresponding to the channels. **C** Diagram of handgrip task. Abbreviations: ROI, region of interest; DH-PFC, prefrontal cortex in the dominant hemisphere; NDH-PFC, prefrontal cortex in the non-dominant hemisphere; DH-PMC, premotor cortices in the dominant hemisphere; NDH-PMC, premotor cortices in the non-dominant hemisphere; DH-M1, primary motor cortex in the dominant hemisphere; NDH-M1, primary motor cortex in the non-dominant hemisphere; DH-S1, primary somatosensory area in the dominant hemisphere; NDH-S1, primary somatosensory area in the non-dominant hemisphere

**Table S1** Comparison of clinical data between post COVID-19 patients and healthy controls

|  | **Patient N=29** | | **Control**  **N=24** | ***P* value** | | |
| --- | --- | --- | --- | --- | --- | --- |
|  | **Patient 1 m** | **Patient 3 m** |  | **1 m vs 3 m** | **1 m vs control** | **3 m vs control** |
| Fatigue measures |  |  |  |  | | |
| ^a^FAS, mean (SD) | 22.9±5.8 | 20.2±5.7 | 19.2±3.5 | **0.019** | **0.006** | 0.425 |
| ^b^FAS＞21 | 16(55.2%) | 10(34.5%) | 6(25.0%) | 0.678 | **0.026** | 0.454 |
| ^a^FSS, mean (SD) | 4.1±1.4 | 3.2±1.5 | 2.8±0.9 | **0.003** | **＜0.001** | 0.251 |
| ^b^FSS≥5 | 11(37.9%) | 3(10.3%) | 1(4.2%) | **0.001** | **0.007** | 0.617 |
| Cardiopulmonary function |  |  |  |  |  |  |
| ^a^6MWT, mean (SD) | 564.028±62.142 | 619.241±79.000^#^ | 602.721±54.161 | **＜0.001** | **0.023** | 0.397 |
| Sleep Quality |  |  |  |  |  |  |
| ^a^PSQI, mean (SD) | 7.2±3.2 | 5.8±2.5 | 5.5±2.7 | **0.008** | **0.044** | 0.678 |
| sleep latency | 1.414±0.983 | 1.241±0.872 | 1.125±1.035 |  |  |  |
| sleep duration | 1.310±0.761 | 1.310±0.660 | 0.917±0.654 |  |  |  |
| sleep efficiency | 0.379±0.775 | 0.207±0.559 | 0.208±0.415 |  |  |  |
| sleep disturbances | 1.310±0.541 | 0.931±0.530 | 0.792±0.509 |  |  |  |
| hypnotic drugs | 0.276±0.591 | 0.069±0.258 | 0.167±0.637 |  |  |  |
| daytime dysfunction | 1.310±1.105 | 1.103±0.944 | 1.083±0.881 |  |  |  |
| ^b^PSQI≥8 | 13(44.8%) | 8(27.6%) | 5(20.8%) | 0.152 | 0.066 | 0.570 |
| Life Quality |  |  |  |  |  |  |
| ^a^EQ-5D-VAS, mean (SD) | 79.6±11.6 | 87.2±7.1 | 88.2±6.5 | **0.001** | **0.002** | 0.626 |
| ^a^EQ-5D Index, mean (SD) | 0.94±0.08 | 0.97±0.03 | 0.97±0.46 | **0.024** | 0.089 | 0.516 |
| Mental health |  |  |  |  |  |  |
| ^c^HAMA, mean (IQR) | 6(1-9) | 2(1-6) | 2(1-6.8) | **0.042** | 0.189 | 0.835 |
| ^b^HAMA≥7 | 13(44.8%) | 6(20.7%) | 6(25%) | 0.052 | 0.134 | 0.709 |
| ^c^HAMD, mean (IQR) | 5(0.5-9) | 3(1-8.5) | 4(1.3-6.8) | 0.455 | 0.653 | 0.971 |
| ^b^HAMD＞7 | 10(34.5%) | 10(34.5%) | 4(16.7) | 1.000 | 0.213 | 0.213 |
| Cognition |  |  |  |  |  |  |
| MMSE, mean (SD) | 30(0) | 30(0) | 30(0) |  |  |  |
| Motor score | 100(0) | 100(0) | 100(0) |  |  |  |

FAS, fatigue assessment scale; FSS, fatigue severity scale; PSQI, pittsburgh sleep quality index; EQ-5D-VAS, euroqol five-dimensional questionnaire visual analogue scale; EQ-5D Index, euroqol health-utility index; HAMA, hamilton anxiety scale; HAMD, hamilton depression scales; 6MWT, 6-minute walking test; MMSE, mini mental-status examination

Significant *P* values are highlighted in bold.

^a^Paired t test or independent sample t-test.

^b^McNemar test or chi-square test

^c^Wilcoxon test or mann-whitney U test

^#^2 patients lost to follow-up were not included in statistical analysis

**Figure S2** Correlation analysis


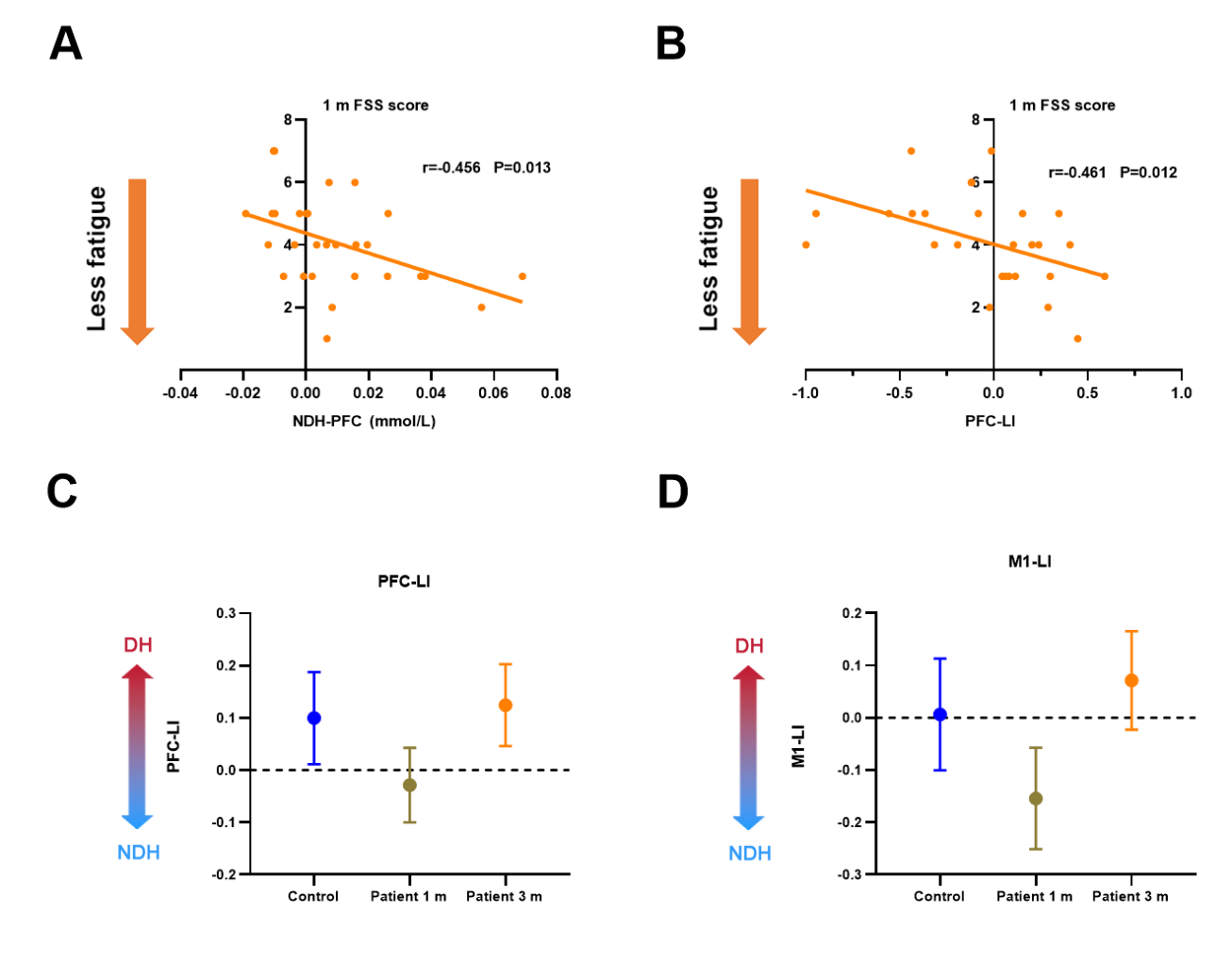


**A** Correlation between activation of NDH-PFC and FSS score at 1 m during left handgrip task. **B** Correlation between PFC-LI and FSS score at 1 m during left handgrip task. **C-D** Comparison of PFC-LI and M1-LI of patients at different time points during right handgrip task. Abbreviations: FSS, fatigue severity scale; PFC, prefrontal lobe; M1, primary motor cortex; LI, laterality index; DH, dominant hemisphere; NDH, non-dominant hemisphere

**Figure S3** Correlation analysis


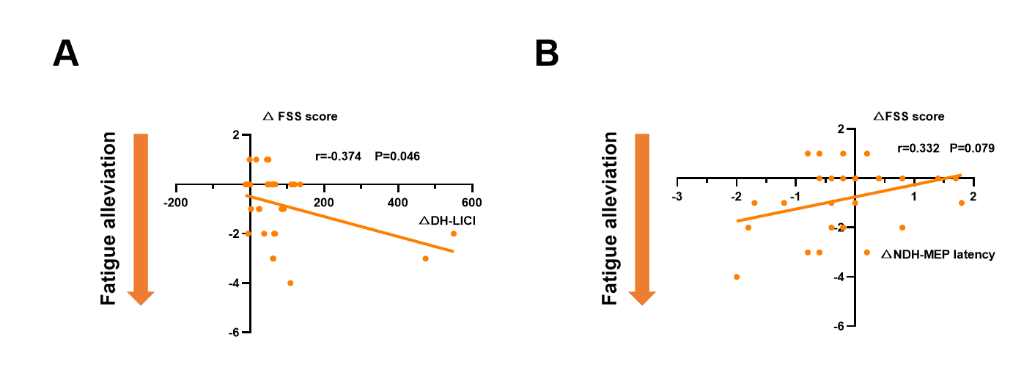


**A** Correlation between DH-LICI change and FSS score change. **B** Correlation between NDH-latency change and FSS score change. Abbreviations: FSS, fatigue severity scale; LICI, long-interval intracortical inhibition; MEP, motor evoked potential; DH, dominant hemisphere; NDH, non-dominant hemisphere

**Table S2** Comparison of baseline data of patients with COVID-19

| **Descriptor** | **Single-infected group N=18** | **Re-infected group^#^**  **N=9** | ***P*** **value** |
| --- | --- | --- | --- |
| Gender, n (%)^a^ |  |  |  |
| Female | 9(50.0) | 7(77.8) | 0.231 |
| Male | 9(50.0) | 2(22.2) |  |
| Age, y, mean (SD)^b^ | 34.8(5.1) | 33.2(9.3) | 0.572 |
| Educational attainment, n (%)^a^ |  |  |  |
| High school or below | 0(0) | 0(0) | 0.435 |
| Junior college degree | 0(0) | 1(11.1) |  |
| Bachelor’s degree | 14(77.8) | 5(55.6) |  |
| Master’s degree | 3(16.6) | 2(22.2) |  |
| PhD | 1(5.6) | 1(11.1) |  |
| Occupation, n (%)^a^ |  |  |  |
| Doctors | 5(27.8) | 2(22.2) | 0.690 |
| Nurses | 6(33.3) | 2(22.2) |  |
| Therapists | 6(33.3) | 5(55.6) |  |
| Other healthcare workers | 1(5.6) | 0(0) |  |

y, year; SD, standard deviation

^a^Exact chi-squared test.

^b^Independent two-sample t-test

^#^Time to re-infection with COVID-19: at 5 m, 2 patients lost to follow-up.

**Figure S4** Comparison between COVID-19 re-infected group and single-infected group


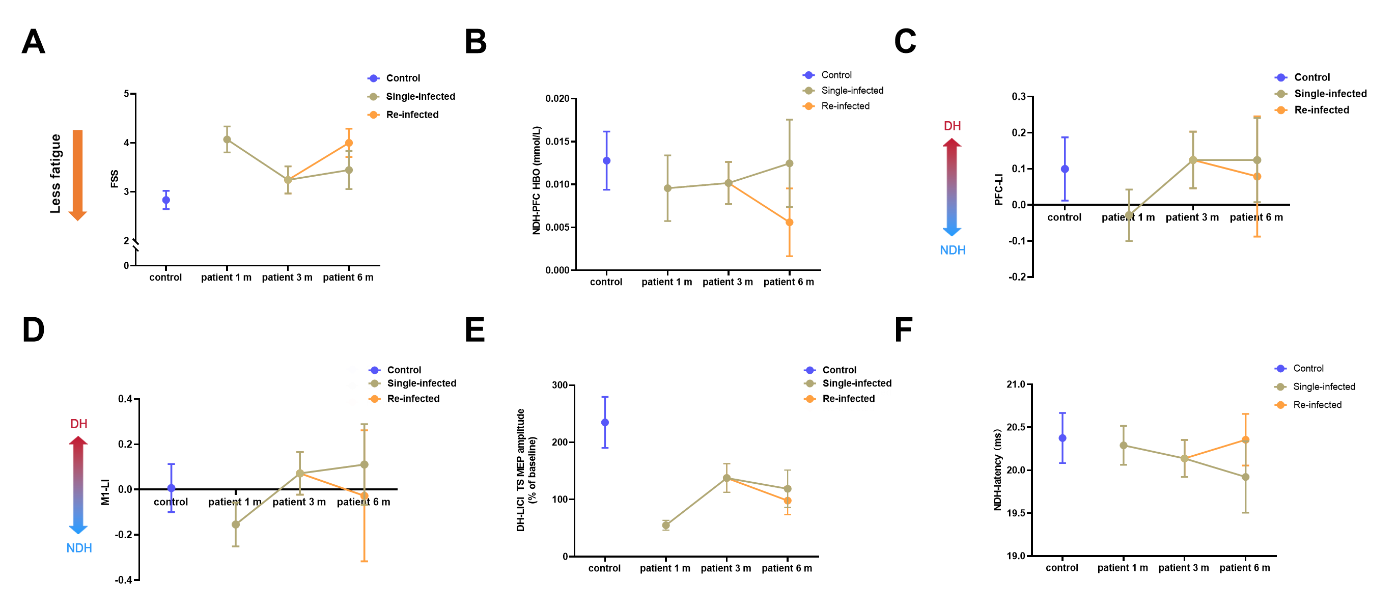


**A** Comparison of FSS between COVID-19 re-infected group and single-infected group at 6 m. **B** Comparison of NDH-PFC between COVID-19 re-infected group and single-infected group at 6 m during left handgrip task. **C** Comparison of PFC-LI between COVID-19 re-infected group and single-infected group at 6 m during right handgrip task. **D** Comparison of M1-LI between COVID-19 re-infected group and single-infected group at 6 m during right handgrip task. **E** Comparison of DH-LICI between COVID-19 re-infected group and single-infected group at 6 m. **F** Comparison of NDH-latency of MEP between COVID-19 re-infected group and single-infected group at 6 m. Abbreviations: FSS, fatigue severity scale; PFC, prefrontal lobe; LI, laterality index; M1, primary motor cortex; LICI, long-interval intracortical inhibition; DH, dominant hemisphere; NDH, non-dominant hemisphere
